# Carbon dot based molecularly imprinted polymer for selective fluorometric determination of tetracycline and metronidazole in pharmaceuticals and human plasma

**DOI:** 10.1038/s41598-025-13474-6

**Published:** 2025-08-08

**Authors:** Khadiga M. Kelani, Maha A. Hegazy, Amal M. Hassan, Ahmed H. Nadim

**Affiliations:** 1 Pharmaceutical Analytical Chemistry Department, Faculty of Pharmacy, Cairo University, Cairo, Egypt; 2 Pharmaceutical Analytical Chemistry Department, Faculty of Pharmacy, Future University in Egypt, 90th St, New Cairo 3, Cairo, Egypt; 3 Pharmaceutical Analytical Chemistry Department, Faculty of Pharmacy, Modern University for Technology and Information, El-Hadaba El-Wosta, Mokatam, 5th district, Cairo, Egypt

**Keywords:** Tetracycline, Metronidazole, Molecular imprinted polymer, Fluorescence, Sensor, Graphene quantum dots, Nanoscale materials, Analytical chemistry

## Abstract

A highly sensitive, selective, and rapid spectrofluorometric method has been developed using fluorescence sensors. This method is based on the highly fluorescent graphene quantum dots coated with silica molecularly imprinted polymers (GQDs-SMIPs) for the determination of tetracycline HCl (TET) and metronidazole (MET). Upon excitation of the GQDs-SMIPs at 260.0 nm for TET and 245.0 nm for MET, strong fluorescence emissions at 292.0 nm were produced for both sensors. Such fluorescence was quenched by the addition of their corresponding molecularly imprinted polymer (MIPs) templates. The quenching effect was linear over the concentration ranges of 15.0–120.0 µM and 15.0–140.0 µM for TET and MET, respectively. Limits of detection (LOD) were 3.55 µM, and 4.48 µM while limits of quantification (LOQ) were 10.75 µM and 13.57 µM for TET and MET, respectively. The fabricated GQDs-SMIPs were characterized using scanning electronic microscopy, Fourier-transform IR spectroscopy, and X-ray diffraction. Selectivity of the method was investigated against potentially interfering substances, including two official impurities of TET and MET. Additionally, this fluorescent technique was successfully applied for the determination of these drugs in pharmaceutical dosage forms and spiked human plasma samples. This approach provides a selective and sensitive fluorometric platform for the determination of the studied drugs in complex matrices and quality control laboratories.

**Supplementary Information:**

The online version contains supplementary material available at 10.1038/s41598-025-13474-6.

## Introduction

Fluorescence sensors have gained significant attention as optical sensing systems with applications in chemical and biological analysis^1–3^. Compared to traditional techniques, fluorescence sensors offer the advantages of being highly sensitive, convenient, rapid, and simple^4,5^. Quantum dots (QDs) are often selected due to their stable photoluminescence, high optical quantum yield, low toxicity, chemical stability and good water dispersity^6^. Fluorescence sensing systems should provide a reliable fluorescent response under the analysis conditions specific to the targeted analyte. Fluorescence sensors are commonly fabricated from carbon-based materials like carbon or graphene due to their two-dimensional structure, which provides a large surface area, high electrical conductivity, and mechanical stability. For this probe, graphene quantum dots (GQDs) were used because they are chemically stabile, less toxic material with stable photoluminescence and large surface area. GQDs have been widely employed in various fluorometric applications^7–9^. Despite the advantageous properties of GQDs-based fluorescence sensors, they lack selectivity. This limitation, however, can be addressed by coating GQDs with molecular imprinted polymer (MIPs)^2^.

MIPs are synthesized using a monomer that co-polymerizes readily with a cross-linker in the presence of a template. After template extraction, the MIP retains 3D cavities with specific recognition sites that match the same shape and size of the template^10,11^. GQDs embedded in silica MIP (GQDs-SMIP) offers the dual advantage of being highly fluorescence and highly selective. GQD-SMIP was synthesized via an eco-friendly sol–gel polymerization using 3-aminopropyltriethoxysilane (APTES) as the functional monomer and tetraethoxysilane (TEOS) as the crosslinker. Sol–gel polymerization has superior advantage over other polymerization techniques as it derived MIPs from rigid silica matrix which resist high temperature and harsh chemical condition better than other traditional MIP. Also, the slow and controlled gelation process provides more stable and precise binding site. In addition, sol–gel utilizes fewer organic solvents which make it more eco-friendly. Such approach emphasizes sustainability throughout the synthesis process by incorporating environmentally friendly principles such as using citric acid—a low-toxicity, biodegradable carbon source—for the pyrolytic synthesis of GQDs, and employing water or ethanol as benign solvents in sol–gel polymerization^12^. The use of APTES and TEOS as silane precursors allows for mild reaction conditions, reducing energy consumption and hazardous byproducts. Additionally, the MIP design offers high selectivity and reusability, minimizing the need for excessive reagents and repeated analyses. These green strategies collectively enhance the environmental compatibility of GQD–SMIPs while maintaining high analytical performance, making them suitable for green environmental sensing applications^13^. Various methods have been reported for synthesizing QD modified MIP such as Mn-doped ZnS QDs^4,14^, GQD and carbon dot (CD) embedded MIP^15,16^ and GQD-coated SMIP^1,16,17^.

Tetracycline HCl (TET), chemically known as (4S,4aS,5aS,6S,12aR)-4-(dimethylamino)-1,6,10,11,12 a pentahydroxy-6-methyl-3,12-dioxo-4,4a,5,5a-tetrahydrotetracene-2-carboxamide hydrochloride, is a broad-spectrum antibiotic that inhibits bacterial protein synthesis^18,19^. Its British Pharmacopoeia (BP) monograph lists four official impurities. Namely, TET impurities A, B, C, and D^20^. Numerous methods for TET determination including fluorometric^21–23^, chromatographic^24–27^, and electrochemical^28–30^ have been documented. Metronidazole (MET); an antibiotic with the chemical name 1-(2-hydroxyethyl)-2-methyl-5- nitroimidazole, inhibits bacterial growth by interfering with nucleic acid synthesis. Seven impurities (A-G) are noted in its BP monograph^20^. Fluorometric^2,31^, chromatographic^27,32,33^, and electrochemical^11,34,35^ techniques have also been reported for its determination.

Co-formulated TET and MET is used to treat stomach and intestinal ulcers caused by *H. pylori* infection. The combined effect of using TET and MET in this formulation is more potent than using each drug alone^18^. Few techniques were reported for the simultaneous determination of the two drugs, including spectrophotometric^19^, chromatographic^18,27^, and potentiometric methods^11^. However, to the best of our knowledge, no fluorometric technique has been reported for their determination.

This study aimed to develop a selective fluorometric method using GQDs coated with MIP for the quantification of TET and MET in complex matrices such as plasma samples. Two specific GQDs-SMIP sensors were fabricated: GQDs-SMIP-TET for TET determination and GQDs-SMIP-MET for MET determination. The selectivity of the fabricated sensors was tested against two official impurities: anhydrotetracycline (TET impurity C) and 4-nitroimidazole (MET impurity B). The structures of the cited drugs are illustrated in (Fig. S1). The developed sensors were also applied for the quantification of TET and MET in spiked human plasma samples. This work represents the first fluorometric approach for the determination of TET and MET using silica MIPs in dosage forms and biological fluids.

## Experimental

### Instruments

Fluorescence spectrometer (model RF-5301PC, Shimadzu, Japan. Excitation wavelengths were set at 260.0 nm for TET and 245.0 nm for MET using slit width of 5.0 nm and scanning rate of 600.0 nm/min. Scanning Electronic Microscope with carbon-coated copper grid (SEM, model JEM-1230, JOEL USA, Inc.). pH glass electrode Jenway analyzer, model 3330 (Essex, UK). IR Spectrophotometer Shimadzu (model 435, Kyoto, Japan). X-ray diffraction (XRD) spectrum (Rigaku, Japan) with a D/max 2500 (18 KW) X-Ray diffractometer.

### Materials and reagents

Pure TET and MET were obtained from Hikma pharmaceuticals (Egypt) with potencies of 99.86 ± 1.274% and 99.26 ± 0.402%, respectively, as assessed according to their BP monographs^36^. MET-impurity B with a certified purity of 100.13 ± 0.960% was purchased from Alfa Aesar (USA). Pylera® capsules (batch no. 3891460505) were labeled to contain 125.0 mg of each of TET and MET per capsule and were manufactured by Allergan (USA). All chemicals used in this study were of analytical grade. Citric acid, sodium chloride, potassium chloride, sodium phosphate dibasic, potassium phosphate monobasic were obtained from Sigma-Aldrich, Germany. Tetraethoxysilane (TEOS) and 3-aminopropyltriethoxysilane (APTES) were sourced from Acros Organics, USA. Methanol, ether, NH_4_OH and glacial acetic acid were supplied by alfa, Egypt, while 36.0% HCl, and NaOH were obtained from El-Nasr, Egypt. Distilled water was obtained from Otsuka (Egypt). Human plasma was purchased from the Holding Company for Vaccines and Biological Products (Vacsera Co), Egypt.

### Preparation of TET impurity C (anhydrotetracycline)

TET official impurity was prepared, as described in our previous work^11^, using acid hydrolysis. In brief, 100.0 mg of TET was dissolved in 100.0 mL methanol and this solution was then transferred to a round bottom flask containing 100.0 mL of 0.1 N HCl. The mixture was refluxed at 40 °C for 7.0 h then neutralized with 0.1 N NaOH. Subsequently, the resulting solution was evaporated to dryness. To ensure complete degradation, the residual powder was analyzed using mass spectrometry. A molecular ion peak at 427 m/z, corresponding to anhydrotetracycline, confirmed complete degradation (Fig. S2). The final solution was reconstituted with methanol to obtain a concentration 1.0 mg mL^−1^.

### Synthesis of graphene quantum dots (GQDs)

Synthesis of GQDs was performed according to previous literature^2^. In a 25-mL beaker, 2.0 g of citric acid was heated to 200.0 °C for 30 min, until citric acid was dissolved and turned to an orange liquid. This liquid was then added dropwise to 100 mL of 10 mg mL^−1^ NaOH with continuous stirring to form GQD solution. The pH of the resulting GQD solution was adjusted to 8.0 using the 10 mg mL^−1^ NaOH. The final GQD solution was stored in the refrigerator for future use.

### Synthesis of GQDs-SMIP

Synthesis of GQDs-SMIP was performed using reported methods^2,16^ where the previously prepared GQDs were entrapped in SMIP using the following procedures. In a beaker containing 16 mL GQDs solution, 5 mmol of APTES was added. The mixture was heated and stirred for 30 min at 40.0 °C. The prepared APTES-modified GQDs (GQDs-APTES) solution was left to cool at room temperature. It was then purified by precipitation with petroleum ether twice and finally dispersed in 40 mL of anhydrous ethanol.

Using typical sol–gel polymerization^2,16^, GQDs were entrapped in silica to form SMIPs. Briefly, 100.0 mg of TET and 100.0 mg of MET (templates) were transferred into two separate 50-mL beakers. To each beaker, 10 mL of GQDs-APTES ethanol solution was added while stirring. Subsequently, 1.0 mL TEOS and 2.0 mL of APTES were added to the solutions. In addition, 2.5 mL of NH4OH were added as a catalyst, and the mixtures were stirred for 30 min^37^. The TET and MET templates were then extracted with methanol: glacial acetic acid solution (90:10*, v/v*) until no templates could be detected in the washing solvent, as verified by UV spectroscopy scanning from 200.0 to 400.0 nm. The solutions were filtered, and the precipitates were dried at 60.0 °C. Non-imprinted polymers embedded with GQDs (GQDs-SNIP) were fabricated using the same procedure mentioned above without the addition of template molecules.

### Characterization of GQDs-SMIP

The fabricated GQDs-SMIP were characterized using multiple techniques to assess their morphology and structure, including infrared spectroscopy (FT-IR), scanning electronic microscope (SEM) and X-ray diffraction spectrum (XRD).

### Optimalization of spectrofluorometric condition

The fluorescence of both GQDs-SMIPs was measured at varying concentrations of their respective templates (TET and MET). Emission scans revealed maximum fluorescence emission at 292.0 nm for both sensors, with excitation at 260.0 nm for TET and 245.0 nm for MET. The excitation and emission slit widths were set to 10.0 nm. Several parameters were investigated to achieve the optimum condition for measurement including amount of GQDs-SMIP used, buffer pH, contact time and the volume ratio between each drug and its corresponding GQDs-SMIP.

Various amounts of GQDs-SMIP (0.01, 0.05, 0.1 and 0.2 g) were tested, and pH values were investigated across a range of pH 4 to 12. Fluorescence was measured at contact time intervals of 15 min up to 75 min. Additionally, the volume ratio between the studied drugs and their corresponding GQD-SMIP solution was optimized using different volume ratios of: 3:1, 2:1, 1:1, 1:2 and 1:3 with fluorescence recorded as described under Section "Optimalization of spectrofluorometric condition".

### Construction of calibration curves

For calibration curve construction, 0.1 g of each prepared GQDs-SMIPs was weighed and suspended in 100-mL volumetric flasks containing 0.1 M phosphate buffer pH 9.0.0 to prepare stocks solutions of GQDs-SMIPs. The flasks were vigorously stirred for 15 min. In two sets of 5-mL volumetric flasks, 2.5 mL of different concentrations of TET standard (30.0–240.0 µM in 0.1 M phosphate buffer pH 9.0) were added to one set, while 2.5 mL of different concentrations of MET standard (30.0–280.0 µM in 0.1 M phosphate buffer pH 9.0) were added to the other set. Each set was completed to volume with the corresponding GQDs-SMIP stock solution previously prepared to obtain a final concentration of TET (15.0–120.0 µM) and MET (15.0–140.0 µM). The mixtures were stirred and incubated for 60.0 min before fluorescence measurements. Furthermore, for selectivity measurements the same procedures were followed except replacing the synthesized GQDs-SMIPs with GQDs-SNIPs.

### Preparation of lab mixtures

Various lab mixtures with different concentrations of the two cited drugs were prepared by transferring different aliquots from the previously prepared TET and MET stock solutions into two sets of 5-mL volumetric flasks. One set was completed to the mark with GQDs-SMIP-TET and the other set was completed to the mark with GQDs-SMIP-MET. The mixtures were thoroughly mixed and incubated for 60.0 min before fluorescence measurements.

### Application to Pylera® capsules

After emptying, mixing, grinding and weighing ten capsules of Pylera®, a powder amount equivalent to one capsule (claimed to contain 125 mg TET and MET) was weighed and dissolved in100-mL volumetric flask containing 0.1 M phosphate buffer pH 9.0 as the diluent. From this solution, 1.0 mL was transferred into 50-mL volumetric flask containing 0.1 M phosphate buffer pH 9.0 as diluent, resulting in final approximate concentration of 56.31 µM for TET and 146.2 µM for MET. Subsequently, 2.5 mL of this diluted solution was transferred into two separate 5-mL volumetric flasks. For TET determination, one flask was completed to volume using GQDs-SMIP specific to TET (flask 1), while for MET determination the second flask was completed to the volume with GQDs-SMIP specific to MET (flask 2). The solutions were then mixed and incubated for 60.0 min before fluorescence measurements.

### Application to human plasma sample

From the previously prepared TET and MET standard solutions in 0.1 M phosphate buffer (pH 9.0), 2.5 mL aliquots were transferred into separate test tubes. To each test tube, 2.5 mL human plasma were added, and the mixture was vortexed for 1.0 min. For protein precipitation, 7.5 mL methanol was added to each tube, followed by vortex for 5 min and centrifugation for 15 min at 4500 rpm. The supernatants were collected, evaporated to dryness using a rotary evaporator, and reconstituted with 2.5 mL of 0.1 M phosphate buffer (pH 9.0). The remaining procedures were carried out as described under Section "Construction of calibration curves".

## Results and discussion

### Synthesis and characterization of GQDs-SMIP

Citric acid pyrolysis was employed for GQDs preparation. It has the advantages of being facile, economic and it did not require complicated processing compared to other raw materials. Upon citric acid melting, the released hydronium ion acts as a catalyst for further decomposition. Through aldol condensation and cycloaddition, aromatic clusters were formed. When these aromatic clusters reach supersaturation point, vigorous nucleation occurs, resulting in GQDs formation^38,39^. For molecular MIP synthesis, silica was selected due to its ease of synthesis, high chemical, physical, and thermal stability, and excellent biocompatibility^40^. In the fabricated GQDs-SMIP, the functional monomer was APTES, a commonly used amino silane silica precursor with the capability to form covalent bonds during the salinization process. TEOS served as the cross-linker and catalytic solution, known for imparting stiffness and acting as a binder.

**Fig. 1:**
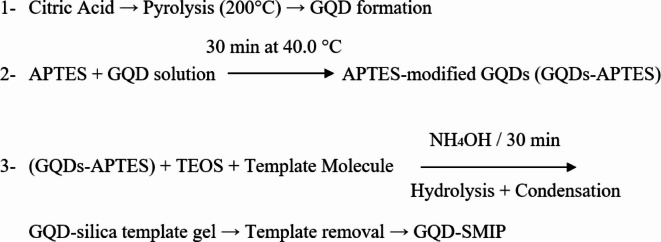
Schematic representation of the synthesis of graphene quantum dots coated with silica molecularly imprinted polymers.

The GQDs-SMIP synthesis involves the following steps: (i) synthesis of GQD from citric acid (ii) GQD were modified to GQD-APTES, where the carboxylic groups of GQDs interacted with the amine group of APTES. GQD alone has a limited surface activity and by modifying with APTES, new reactive sites were added to make it ready for sol–gel polymerization step. APTES also formed hydrogen bond between its amino groups and functional groups in the template molecules creating a pre-polymer. (iii) Salinization of the pre-polymer which immobilize it on the GQDs surface. APTES (monomer) and TEOS (crosslinker) underwent simultaneous hydrolytic condensation coating the GQDs with SMIP via sol–gel polymerization^2,16^. After templates extraction using a methanol: acetic acid (9: 1, v/v) solution, two GQD-SMIP variants were obtained: GQD-SMIP- TET (for TET) and GQD-SMIP- MET (for MET). The GQDs-SMIP synthesis was summarized in Fig. 1.

The GQDs-SNIP served as controls to evaluate the success of the molecular imprinting process, by comparing their characteristics with those of the fabricated MIPs. SEM analysis revealed that, after template removal, the MIP exhibited a more porous surface morphology than the NIPs, indicating effective template imprinting. FTIR spectroscopy further supported these findings. While the NIP shared similar structural features with the MIP, the imprinted polymer displayed more pronounced absorption bands. This enhancement is attributed to the formation of hydrogen bonds and electrostatic interactions between the MIP and its template molecule, confirming successful imprinting. Notably, the broader and more intense N–H stretching vibration at 3383 cm^−1^ in the MIP spectrum, reflected enhanced hydrogen bonding among the GQDs, the template, and the polymer matrix. Additionally, the slight increase in the Si–O–Si stretching band near 1100 cm^−1^ observed in the MIP, suggested more complete condensation of the siloxane network around the template. These differences confirm the successful formation of specific recognition sites in the MIP.

#### Fourier-transform infrared (FT-IR)

The FT-IR absorption spectra of GQDs-SMIP-TET, GQDs SMIP-MET and GQDs-SNIP showed characteristic stretching peaks at 1056 cm^-1^ (Si–O–C) and 1145 cm^−1^ (Si–O–Si), indicating efficient silica attaching to GQDs, as presented in (Fig. S3)^2^. Additionally, stretching peaks around 460 cm^−1^ and 790 cm^−1^ confirmed the presence of the Si–O vibrations. A peak at 1640 cm^−1^ corresponded to the amide group (–CO–NH–) while broad stretching peaks around 3383 cm^−1^ and 2943 cm^−1^ were attributed to N–H bond and C–H bonds, respectively. It is noteworthy that the fabricated GQDs-SNIP had shared similar structural features with GQDs-SMIP. However, the peaks were more pronounced in the imprinted GQD-SMIP (compared to the corresponding GQDs-SNIP), owing to creation of hydrogen bonds and electrostatic interactions between the MIP and its related drug^41^, reflecting the effectiveness of the imprinting process. N–H stretching (3383 cm^−1^) in SMIP exhibited a broader and more intense band due to hydrogen bonding between GQDs, template, and polymer matrix. In GQD-SMIP, more complete condensation (more Si–O–Si) can occur around the template, slightly increasing the band intensity at 1100 cm^-1^ (Fig. S3). Increased peak intensity in MIP for functional groups associated with the template suggests successful imprinting and specific binding site formation.

#### Field-emission scan electronic microscope (FE-SEM)

**Fig. 2:**
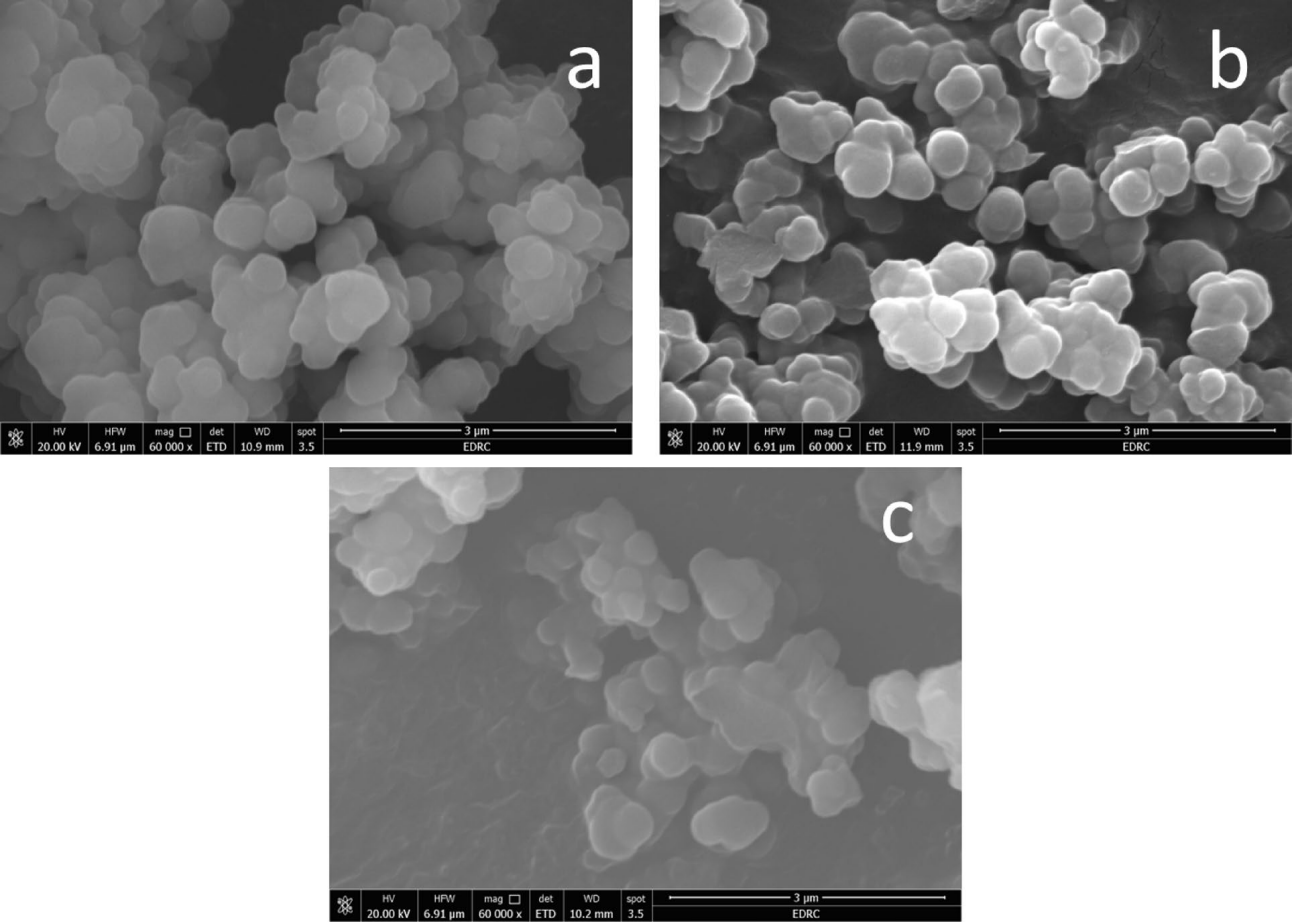
SEM images for the proposed GQDs-SMIPs of (**a**) TET, (**b**) MET, and (**c**) their corresponding NIPs at a magnification power of 6 × 10^4^.

The surface morphology of the fabricated GQDs-SMIPs and the corresponding GQDs-SNIP was characterized using FE-SEM. As shown in Fig. 2, the particles exhibited spherical shape with an average diameter of approximately 50.0 nm. Structural differences between the prepared MIP and NIP were evident; after templates removal the synthesized MIP appeared more porous, reflecting the imprinting effect.

#### X-ray diffraction spectrum (XRD)

The XRD patterns of both GQDs-SMIPs and GQDs-SNIP showed one broad peak at approximately ~ 21°, attributed to the silica polymer on the GQDs particles. Additionally, a wide peak around ~ 24° corresponds to (002) plane of GQDs. Peak broadness could be attributed to the amorphous nature of silica (broad background) and embedding within a heterogeneous polymeric structure. In general, SMIPs show lower overall crystallinity than pure GQDs. In addition, template extraction can lead to porosity, which can cause further disordering in the polymer matrix as shown in Fig. S4^1,42,43^.

### Absorption and photoluminescence properties of GQDs-SMIP

**Fig. 3:**
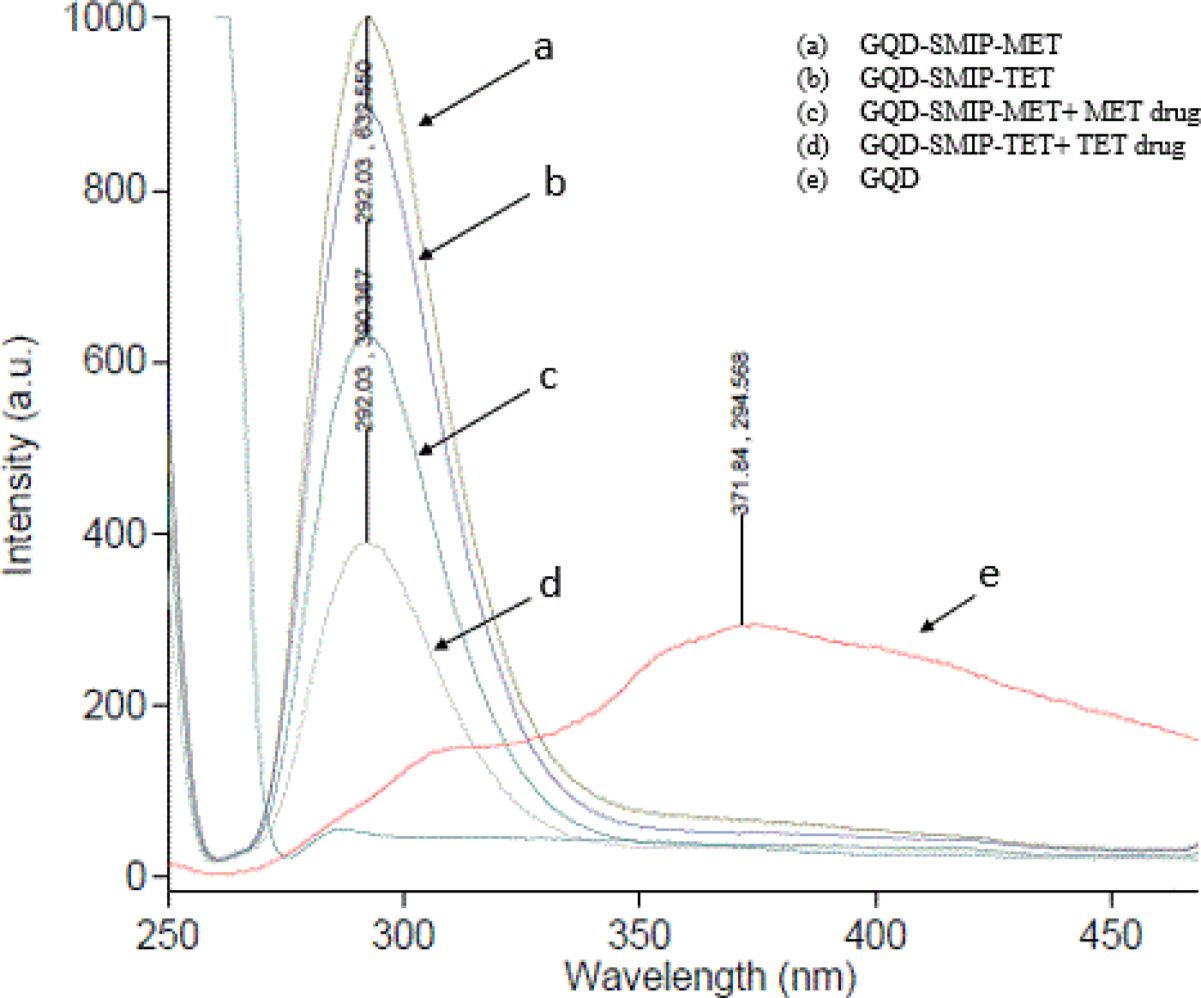
Emission spectra of (**a**) GQD-SMIP-MET, (**b**) GQD-SMIP-TET, (**c**) GQD-SMIP-MET + MET drug, (**d**) GQD-SMIP-TET + TET drug, and (**e**) GQD.

When excited at 260.0 nm and 245.0 nm for TET and MET, respectively, both GQDs-SMIP variants exhibited an emission fluorescence peak at 292.0 nm. The fabricated fluorescent GQDs-SMIP-TET and GQDs-SMIP-MET effectively quantified TET and MET, respectively. As presented in Fig. 3, the emission spectrum of GQDs was changed after modifying it with MIP to form GQDs-SMIP. MIP interacted with the surface of GQDs followed by altering the surface function groups. After template removal, the GQDs-SMIPs exhibited strong fluorescence, attributed to surface passivation and structural isolation within the porous silica matrix. However, upon interaction with their corresponding drug molecules, fluorescence intensity was significantly quenched. This is consistent with findings of Han et al.^44^ who attributed such fluorescence quenching of the GQDs-SMIPs to electron transfer between the GQD and the drug molecules. In GQD-SMIP, template rebinding places the drug molecules to close spatial proximity to the embedded GQDs enabling photoinduced electron transfer (PET). After template removal, the GQDs exhibit strong fluorescence, supported by surface passivation and structural isolation within the porous silica matrix. When the template is reintroduced, it selectively binds into the imprinted cavities near the GQDs, facilitating photoinduced electron transfer (PET). This interaction disrupts the excited-state recombination of GQDs, resulting in pronounced fluorescence quenching. This behavior not only confirms the successful imprinting of recognition sites but also highlights the potential of GQD-SMIP as a sensitive fluorescence-based sensing sensors^42^.

In conclusion, the fluorescent emission of GQDs-SMIPs decreased proportionally with increasing concentrations of the target drug. The binding efficiency of the prepared GQD-SMIP was demonstrated in terms of imprinting factor (IF). GQDs-SNIP samples were analyzed under the same conditions as the GQDs-SMIPs—both alone and after the addition of TET and MET at their respective excitation wavelengths, using the proposed fluorescence method. The only change was the substitution of GQDs-SMIPs with GQDs-SNIP for the purpose of imprinting factor calculation. The fluorescence quenching obtained from both MIP and NIP was calculated and used to evaluate IF as follows: IF = response of GQDs-SMIP/response of GQDs-SNIP. The IF was calculated as the ratio of the template adsorbed on same polymer with and without implementing the imprinting procedure. A relatively high IF of 7.2 was achieved, indicating efficient binding and selectivity of the imprinted polymer.

The effect of pH was investigated by adjusting the pH of 0.1 M phosphate buffer from 4.0 to 12.0 and calculating the fluorescence ratio (F_0_/F) where, F_0_ is the fluorescence intensity of the fabricated GQD-SMIP before adding its related drug, while F represents the fluorescence intensity of GQD-SMIP after drug addition. As the pH increased from 4.0 to 9.0, the fluorescence quenching by the drugs increased, likely due to the protonation of the GQDs-SMIP binding sites at lower pH decreasing their interaction with the template. Conversely, at pH levels above 9.0, the fluorescence ratio had decreased, suggesting that the imprinted cavities could have been damaged or hydrolyzed. Therefore, pH 9.0.0 was selected as the optimum pH for TET and MET determination (Fig. S5). The amount of GQDs-SMIP used in preparing stock solutions was also optimized and it was found that 0.1 g of GQDs-SMIP yielded the maximum fluorescence intensity. Increasing the amount to 0.2 g, the fluorescence intensity was not detected by the instrument. On the other hand, upon decreasing the amount to 0.01 or increasing it to 0.5 g lower fluorescence intensities were observed confirming that, 0.1 g GQDs-SMIP was the ideal amount.

The effect of contact time between the GQD-SMIP and its corresponding drug was also examined, with contact time ranging from 15 to 60 min. Results showed that longer contact times led to increased fluorescence quenching, reaching a maximum at 60 min. Extending contact time beyond 60 min did not further increase the quenching effect, indicating that 60 min was sufficient. Additionally, the effect of the volume ratio between the GQD-SMIP solution and its corresponding drug on the fluorescence and quenchingwas studied. Various volume ratios were tested: 3:1, 2:1, 1:1, 1:2 and 1:3. Optimal fluorescence and quenching effects were achieved with equal volumes of GQDs-SMIP solution and its corresponding drug (1:1 ratio), as shown in Fig. S6a and b.

### Fluorescence detection of TET and MET using GQDs-SMIPs

**Fig. 4:**
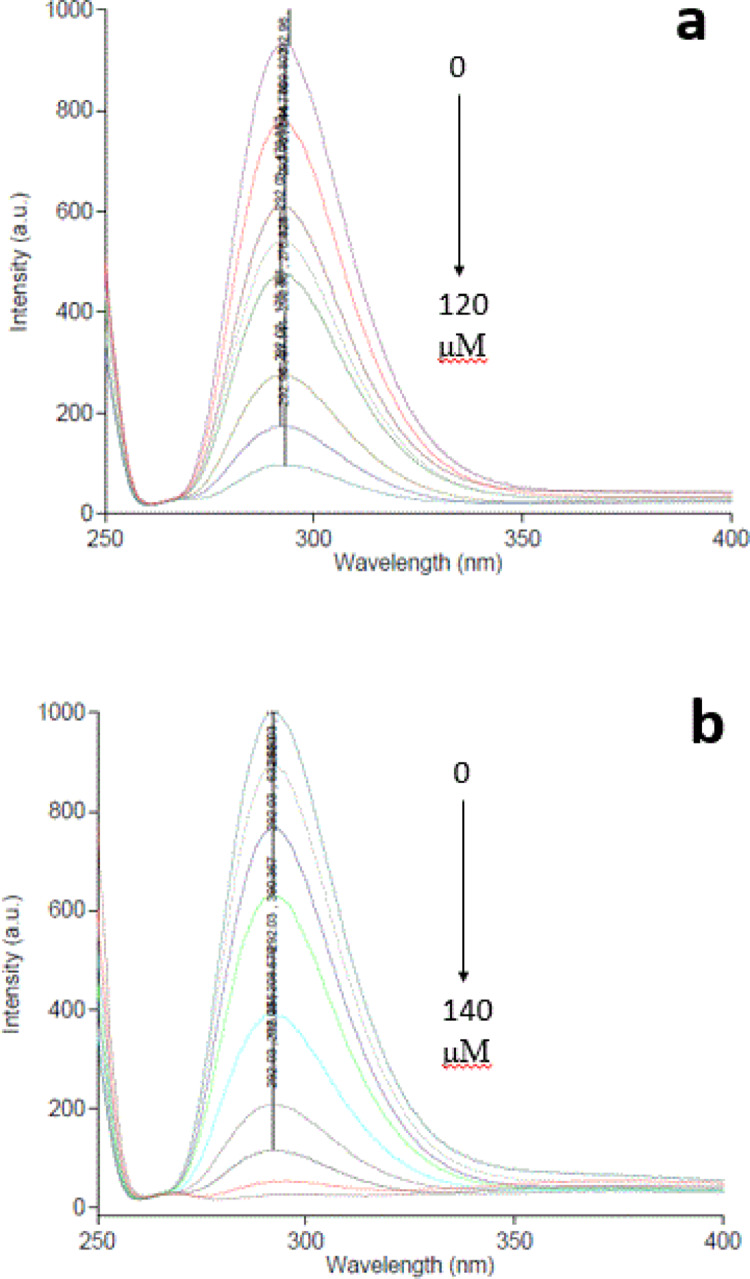
Emission spectra of GQDs-SMIP at different concentrations of (**a**) TET and (**b**) MET.

The fluorescence intensities of GQD-SMIPs of TET and MET were quenched upon addition of their corresponding drug due to hydrogen bond formation between the templates and the amine groups on the surface of GQDs-SMIP^45^. Notably, the behavior of GQD-SNIP differed from GQDs-SMIP. As the concentration of the corresponding template increased, the fluorescence intensity of GQD-SMIPs was clearly quenched, while the fluorescence intensity of GQDs-SNIP showed minimal change in fluorescence intensity. When the concentration of TET and MET was raised from 15.0 to 120.0 µM and 15.0 to 140.0 µM, respectively, the fluorescence intensity of GQDs-SMIP-TET and GQDs-SMIP-MET was quenched, as shown in (Fig. 4a,b).

### Method validation

A linear relationship was observed for TET and MET within the concentration ranges of 15.0–120.0 µM and 15.0–140.0 µM, respectively, after plotting the fluorescence intensities ratios against their respective concentrations. The limits of detection (LOD), limits of quantification (LOQ), relative standard errors, and regression equations were summarized in Table 1. Linear relationships with R^2^ values of 0.9993 for TET and 0.9992 for MET were achieved, correlating the fluorescence intensity ratio (F_0_/F) at an emission wavelength of 292.0 nm with varying concentrations of TET or MET (Fig. S7a and b) with LOD of 3.55 for TET and 4.48 µM for MET. This fluorometric technique, utilizing GQDs embedded in silica MIP, was established for the first time for the determination of TET and MET. Accuracy was assessed using three different concentrations of each drug (30.0, 60.0, 90.0 µM), with the calculated recoveries presented in Table 1. Three concentrations (30.0, 60.0, 90.0 µM) were selected for TET and MET from their linearity range. Each concentration was determined three times within the same day to assess intra-day precision. The calculated RSD % values were summarized in Table 1. Moreover, intermediate precision was evaluated by analyzing the previously selected three concentrations three times across three days to assess inter-day precision. The calculated RSD % was presented in in Table 1. Finally, robustness was assessed by making small changes in the analytical condition and measuring its effect on the quenching effect. The changes performed were the reaction time (60.0 ± 1.0 min), and pH (9.0 ± 0.1). The RSD % was shown in Table 1.

**Table 1:** Regression and validation parameters for determination of tetracycline and metronidazole using the proposed Method.

Parameters	TET	MET
Linearity range (µM)	15.0–120.0	15.0–140.0
Slope	0.0742	0.1419
Intercept	0.6272	-0.0321
Correlation coefficient (r)	0.9996	0.9995
SE of the slope	0.0010	0.0020
SE of the intercept	0.0654	0.1489
Accuracy (recovery %)^a^	99.71	100.05
Precision (RSD %)		
Repeatability^b^	1.03	1.48
Intermediate precision^c^	1.82	1.89
Robustness (RSD%)^d^	1.46	1.73
LOD (µM)^e^	3.55	4.48
LOQ (µM)^e^	10.75	13.57

^a^Average of nine determinations (three different concentration three times each).

^b^RSD% of three different concentration three times within the same day.

^c^RSD% of three different concentration three times (across three days).

^d^Studied factors: reaction time (60.0 ± 1.0 min), and pH (9.0 ± 0.1).

^e^LOD = 3.3 σ/ S, LOQ = 10 σ/ S :- where, σ is the SD of the response and S is the slope.

### Selectivity

**Fig. 5:**
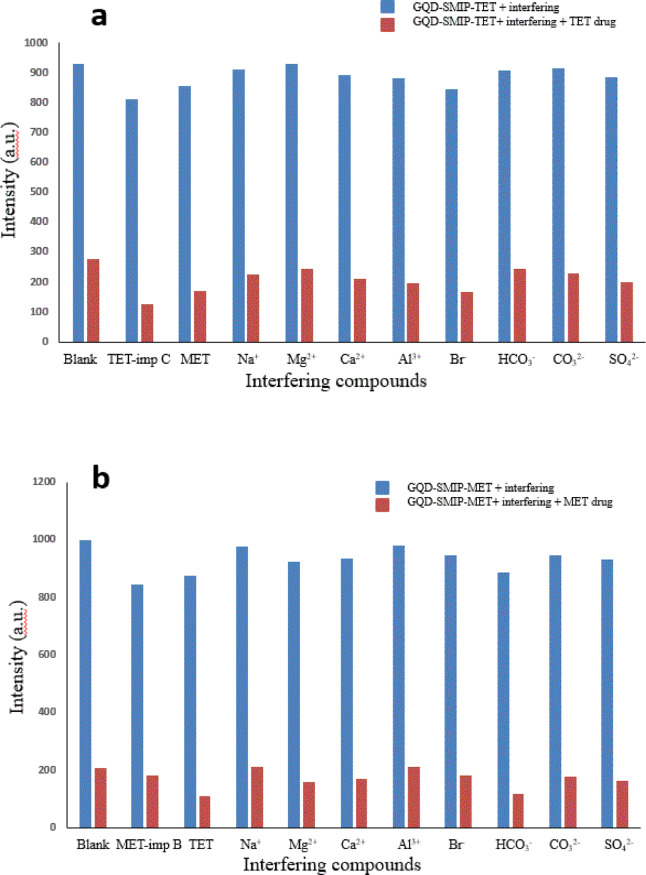
Fluorescence intensity of (**a**) TET and other species in relation to GQDs-SMIP-TET, and (**b**) MET and other species in relation to GQDs-SMIP-MET.

Selectivity of the two synthesized sensors was evaluated using prepared laboratory mixtures. The selectivity of GQD-SIMP-TET was assessed against a structurally related compound (TET-impurity C) and the co-formulated drug MET, while the selectivity of GQD-SIMP-MET was tested against a structurally related compound (MET-impurity B) and its co-formulated drug TET. Additionally, the method’s robustness was tested with various potential interfering ions, including metal cations (Na⁺, Ca^2+^, Mg^2+^, and Al^3+^) and anions (HCO_3_^−^, Br^−^, SO_4_^2−^, and CO_3_^2−^), under previously described conditions (Fig. 5a,b). Results indicated that the GQD-SMIPs were highly selective for their target drugs, with minimal response observed from interfering molecules. This selectivity is attributed to the specific cavities in the molecularly imprinted silica polymer, which match the shape and size of the template molecule.

### Stern–Volmer plot

The Stern–Volmer equation is used in fluorescence determination for describing quenching. The equation gives indication of how much the quencher affect quenching and which type of quenching occur either dynamic or static. And this could be known by calculating Stern–Volmer quenching constant (K_SV_) from the following equation:

$${\text{Io}}/{\text{I}} = 1 + {\text{K}}_{{{\text{SV}}}} \left[ {\text{Q}} \right]$$

where Io: the fluorescence intensity without quencher, I: the fluorescence intensity with quencher, K_SV_: Stern–Volmer quenching constant, and Q: the concentration of the quencher.

Stern–Volmer plots were obtained (Fig. S8) using TET and MET as a quencher separately and from those curves we found that the plots between Io/I versus Q are linear indicating dynamic quenching effect. Moreover, the obtained K_SV_ were 72.63 M^−1^ and 135.8 M^−1^ for TET, and MET, respectively. The large value for the obtained K_SV_ indicated the more efficient quenching^46^.

### Analytical applications

The proposed fluorometric method successfully determined TET and MET in Pylera® capsules without interference from capsule excipients, as presented in Table 2. The method was also applied to spiked human plasma samples containing TET and MET, yielding high recoveries (Table 3). Comparing this study to other published methods for TET and MET analysis^19,27,28,47–51^, the proposed method offers a simpler fluorescence-based approach that provides comparable results to those obtained by sophisticated techniques which often require costly equipment and complex procedures, as illustrated in Table 3.

**Table 2:** Determination of tetracycline and metronidazole in dosage forms and spiked human plasma samples using the proposed method.

Application	TET	MET
	Found % ± SD^a^	
Pylera ® capsule Labelled to contain 125.0 mg of TET and MET per capsule	99.26 ± 0.88	101.70 ± 1.97
Spiked plasma samples (µM)	Recovery % ± SD	
30.0	87.31 ± 1.48	83.80 ± 0.87
60.0	81.69 ± 1.63	83.82 ± 0.92
90.0	86.32 ± 2.53	85.99 ± 1.47

^a^The recovery and found percentages are the average of three determinations.

**Table 3:** Recently reported methods for the determination of tetracycline and metronidazole.

Ref. No	Analyte to determine	Linearity range	LOD	Method of determination	Application
^27^	TET MET	2.0–40.0 µg mL^−1^ 2.0–40.0 µg mL^−1^	0.61 µg mL^−1^ 0.60 µg mL^−1^	Solid phase extraction coupled with HPLC	Pharmaceutical dosage form Spiked human plasma
^28^	TET MET	1.0 × 10^−7^–1.0 × 10^−2^ M 1.0 × 10^−6^–1.0 × 10^−2^ M	5.88 × 10^−8^ M 5.19 × 10^−7^ M	Electrochemical	Pharmaceutical dosage form Spiked human plasma
^47^	TET MET	12.5–62.5 µg mL^−1^ 12.5–62.5 µg mL^−1^	NA NA	HPLC	Pharmaceutical dosage form
^19^	TET MET	12.5–62.5 µg mL^−1^ 12.5–62.5 µg mL^−1^	NA NA	UV-spectroscopy	Pharmaceutical dosage form
^48^	TET	10.0–400.0 µM	6.0 µM	Fluorimetry based on CD technique	Pharmaceutical dosage form
^49^	TET	0.0–20.0 µM	8.2 nM	Fluorimetry based on GQDs-Eu^3+^ technique	River water Milk sample
^50^	MET	7.5 × 10^−4^–2.5 × 10^−8^ g mL^−1^	1.08 × 10^−10^ g mL^−1^	Fluorimetry based on ZnO-doped CQDs technique	Pharmaceutical dosage form
^51^	MET	0.0–10.0 µg mL^−1^	0.257 µg mL^−1^	Fluorimetry based on CD technique	Pharmaceutical dosage form Honey sample
This work	TET MET	15.0–120.0 µM 15.0–140.0 µM	3.55 µM 4.48 µM	Fluorimetry based on GQD-SMIP technique	Pharmaceutical dosage form Spiked human plasma

### Statistical analysis and comparison with other reported methods

Results obtained for TET and MET using the proposed method were statistically compared to those obtained from their official methods^20^. No significant difference was observed indicating the strong applicability of the proposed sensors (Table 4).

**Table 4:** Statistical comparison between the results obtained using the proposed method and those from the official method.

Parameter	TET	MET	Official method 20for TET	Official method^20^ for MET
Mean of recoveries	99.71	100.05	99.86	99.26
SD	1.160	0.736	1.272	0.399
Variance	1.345	0.541	1.619	0.160
n	5	5	5	5
Student’s *t*-test	0.195 (2.306)	2.121 (2.306)	NA	NA
*F*-test	1.203 (6.388)	3.393 (6.388)	NA	NA

^a^The values in parentheses represent the corresponding tabulated values of t and F at *p* = 0.05.

## Conclusion

This study introduces a selective fluorometric approach for the determination of TET and MET in pharmaceutical formulations and spiked human plasma samples. Two fluorometric sensors were developed using GQD coated with the corresponding molecular imprinted polymer, enhancing the selectivity of GQD alone. The synthesized sensors were characterized using IR, SEM and XRD techniques. The selectivity of the sensors was assessed against the official impurities TET impurity-C and MET impurity-B. The proposed method successfully determined the two drugs in their co-formulated dosage forms and in spiked human plasma samples.

## Supplementary Information

Below is the link to the electronic supplementary material.

Supplementary Material 1

## Data Availability

The datasets used and/or analysed during the current study are available from the corresponding author on reasonable request.
