# Supplementary material for: Carbon dot based molecularly imprinted polymer for selective fluorometric determination of tetracycline and metronidazole in pharmaceuticals and human plasma
Source: Sci Rep. 2025 Aug 8;15:29039. doi: 10.1038/s41598-025-13474-6 (PMC12334689; doi:10.1038/s41598-025-13474-6)
Supplement: Supplementary file 1 — Supplementary Material 1 [file 41598_2025_13474_MOESM1_ESM.docx]

Supplementary material

**Figure S1**

**
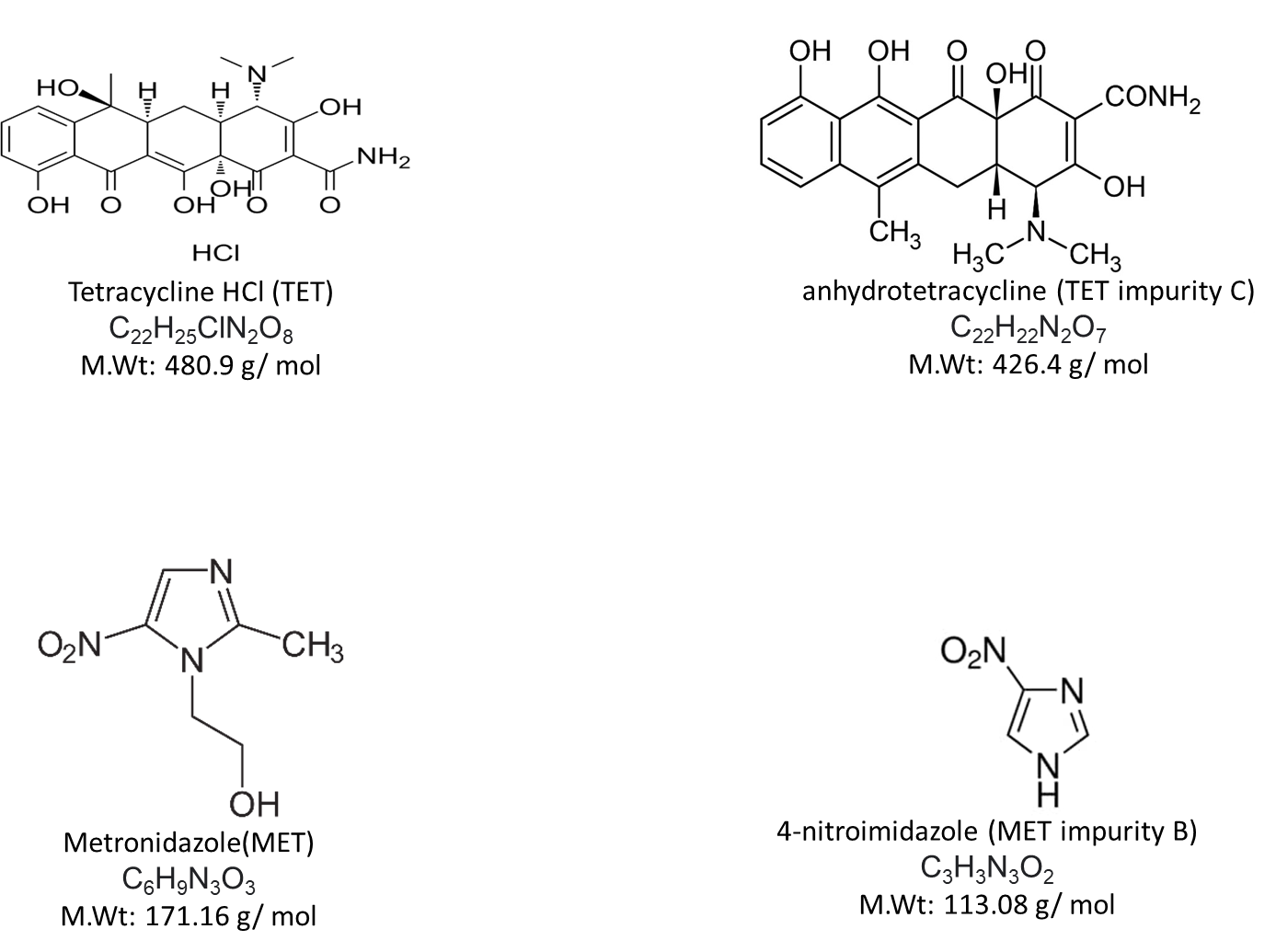
**

**Figure S1.** Chemical Structure of the studied compounds.

**Figure S2**


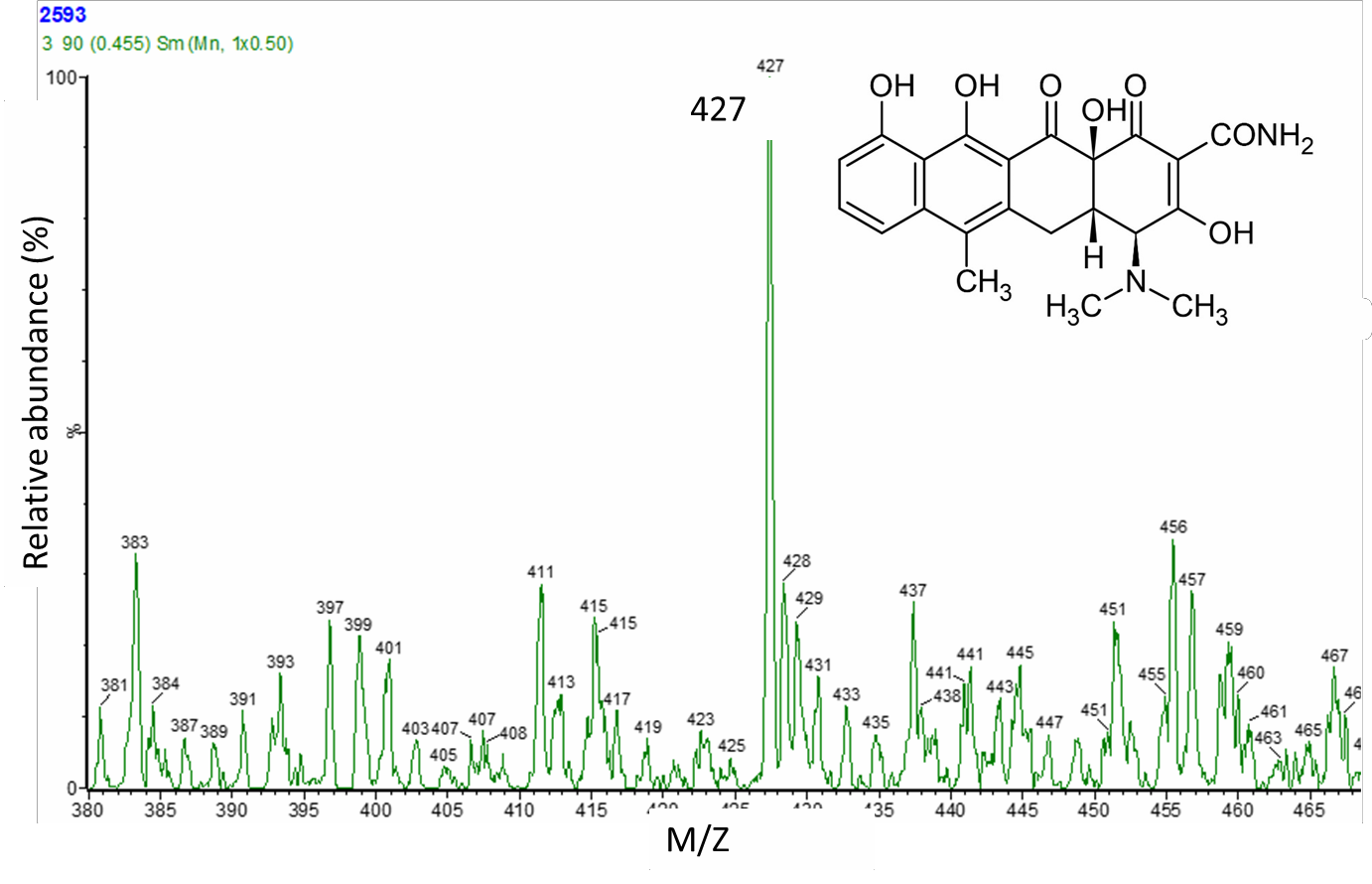


**Figure S2**. Mass spectrum of anhydrotetracycline (TET imp-C) displaying its molecular ion peak at 427 m/z.

**Figure S3**

**
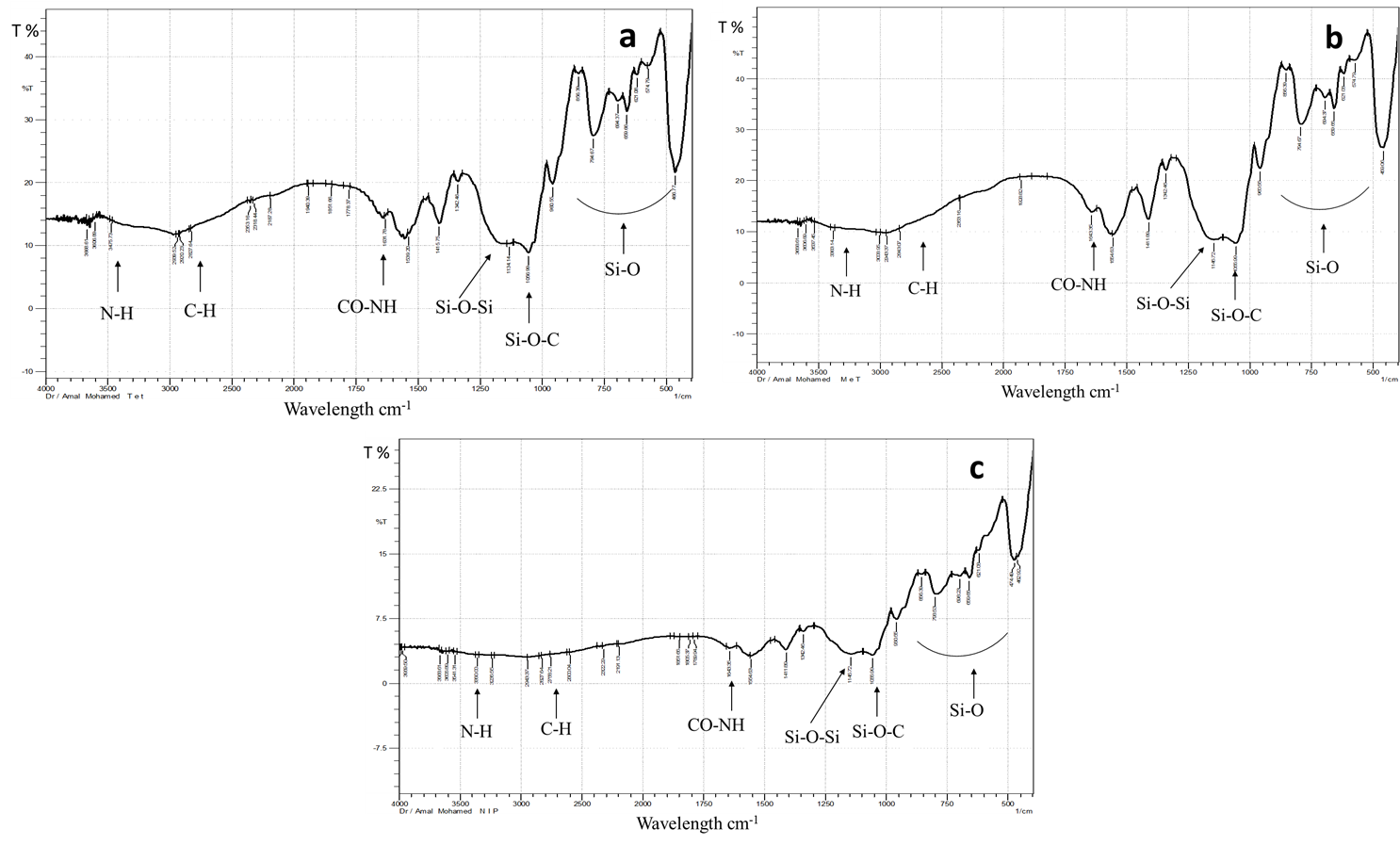
**

**Figure S3.** FTIR spectra for the proposed GQDs-SMIPs of (a) TET, (b) MET and (c) their corresponding NIPs.

**Figure S4**


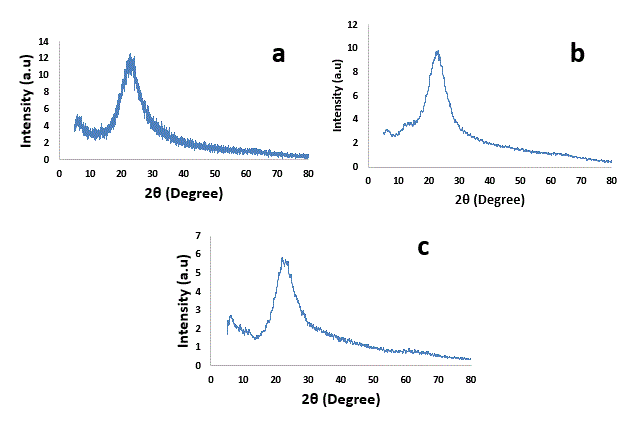


**Figure S4.** The obtained XRD spectra for (a) GQDs-SMIP-TET, (b) GQDs-SMIP-MET, and (c) their corresponding NIPs.

**Figure S5**


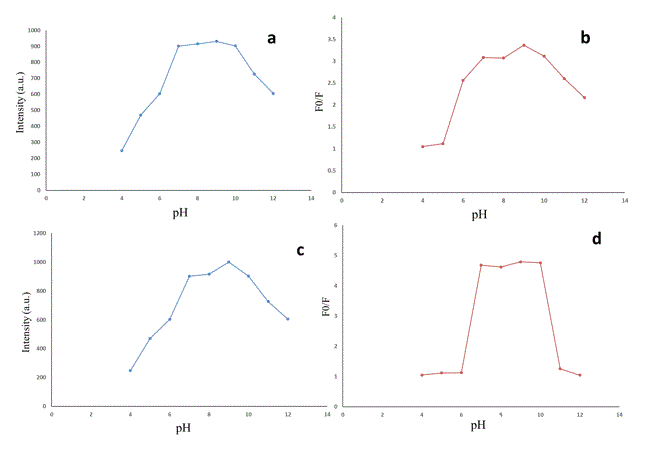


**Figure S5**. Fluorescence intensity of (a) GQDs-SMIP-TET, (b) quenching effect with 35.0 μM of TET drug, (c) GQDs-SMIP-MET, and (d) quenching effect with 35.0 μM of MET drug at various pH values

**Figure S6**


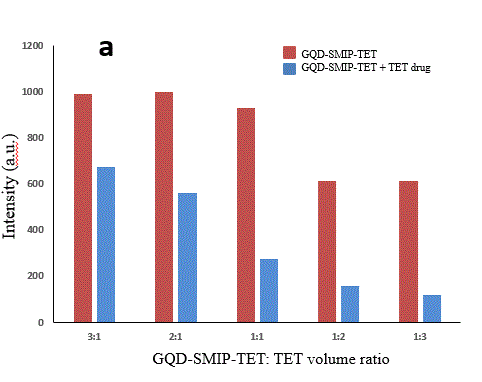

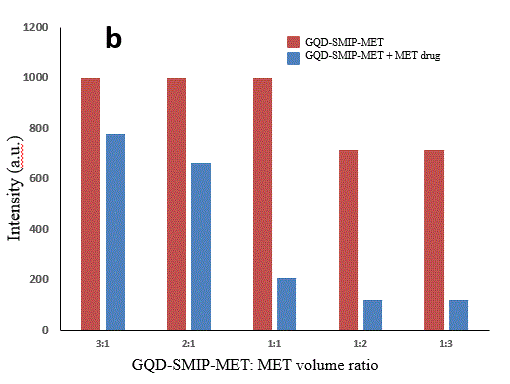


**Figure S6.** The effect of volume ratio on the fluorescence intensity of (a) GQD-SMIP-TET for TET and (b) GQD-SMIP-MET for MET

**Figure S7**

**
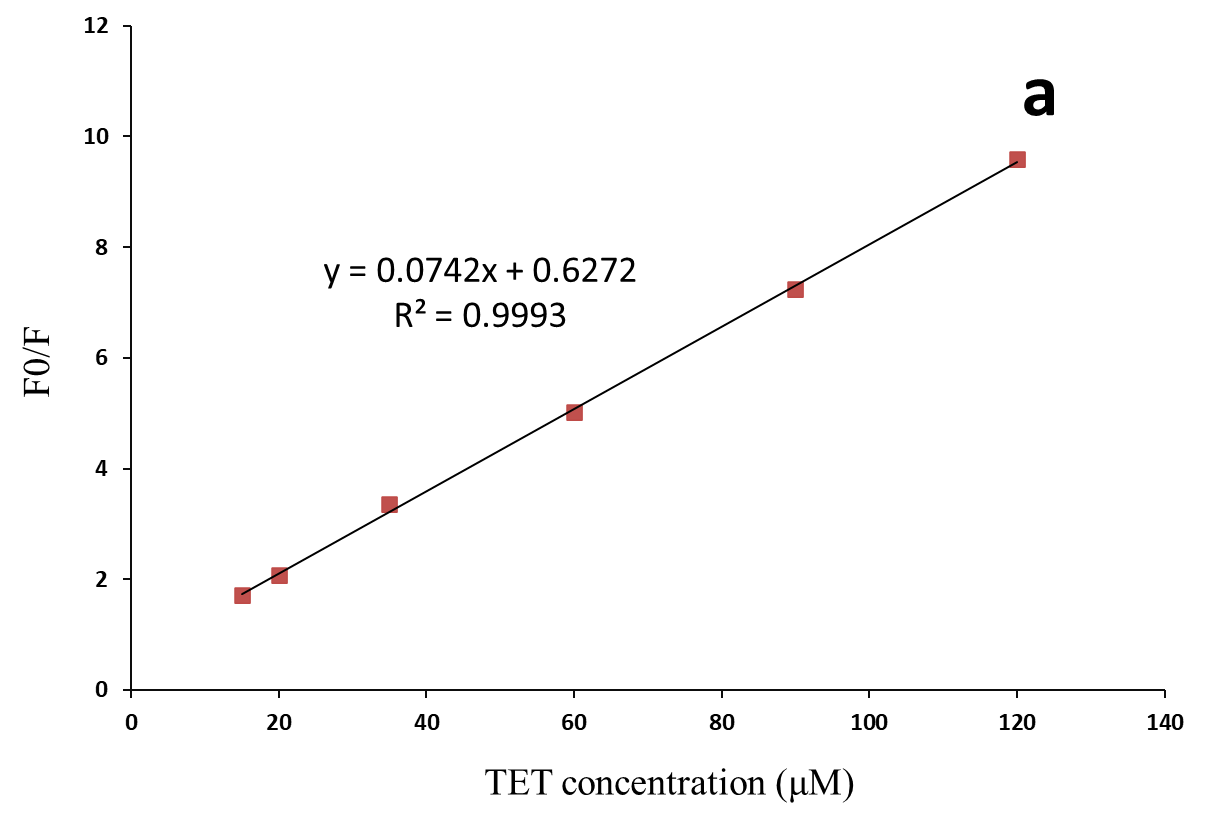

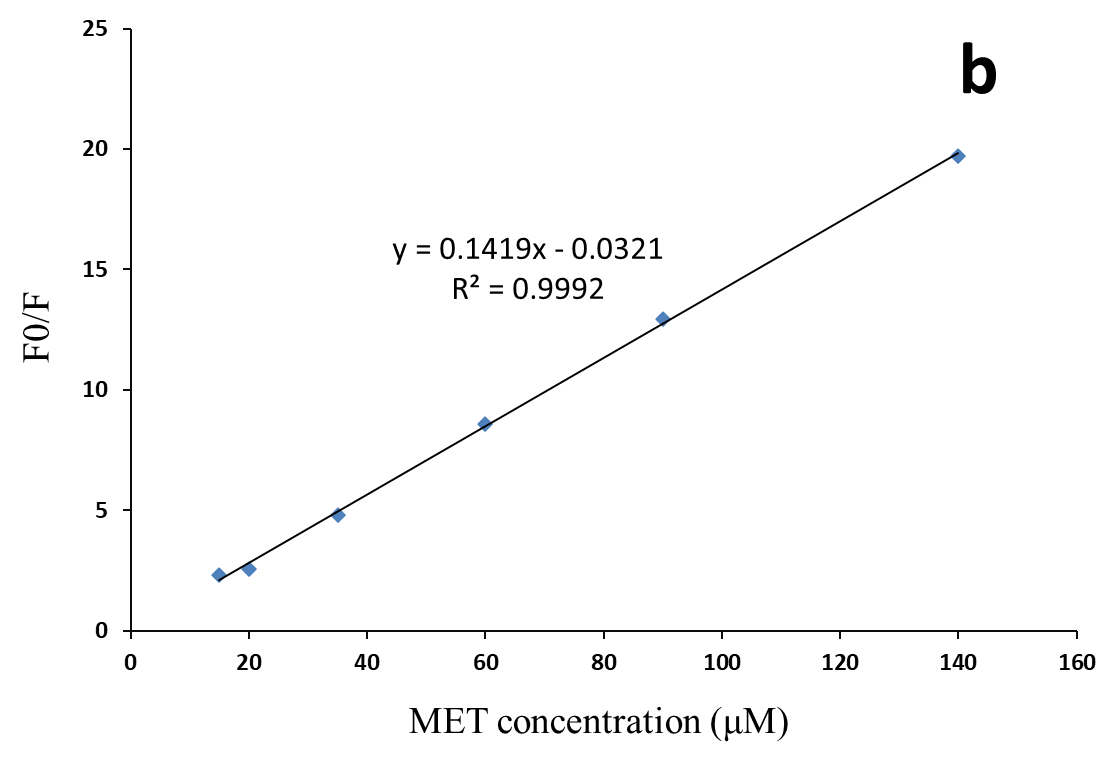
**

**Figure S7.** Linear relationship between F₀/F and (a) TET concentration, and (b) MET concentration.

**Figure S8**


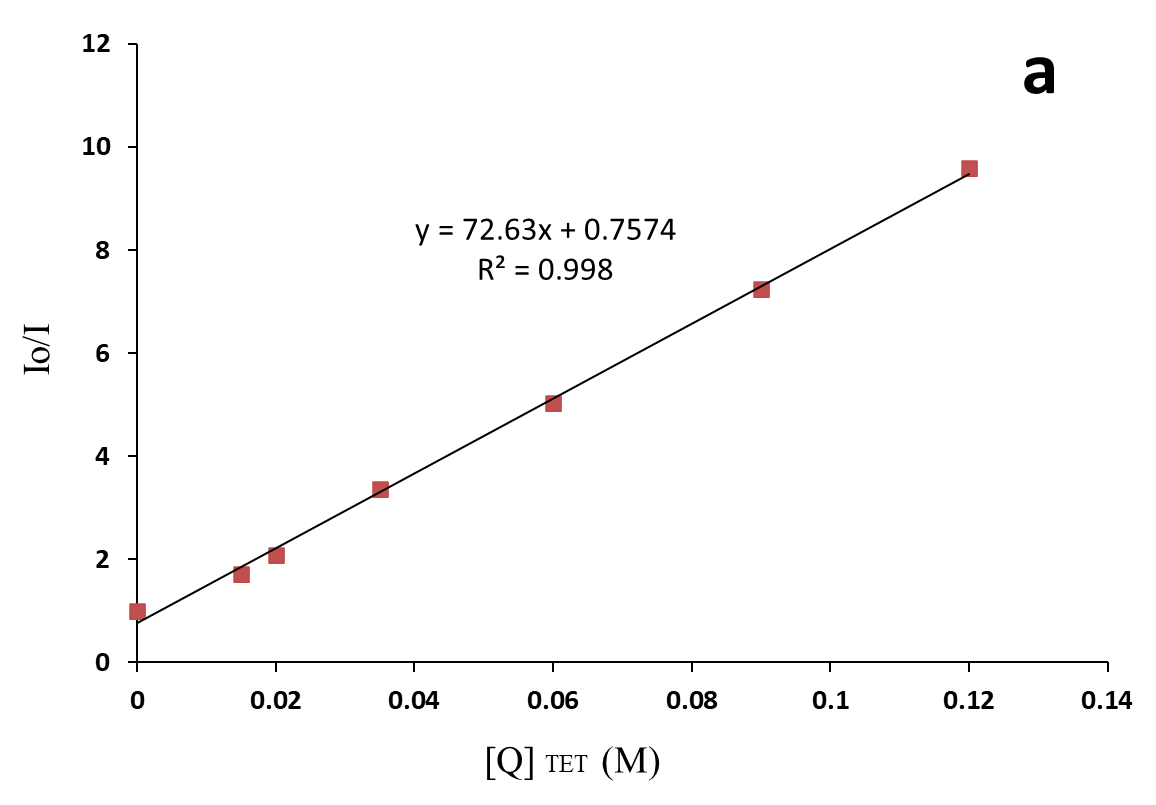

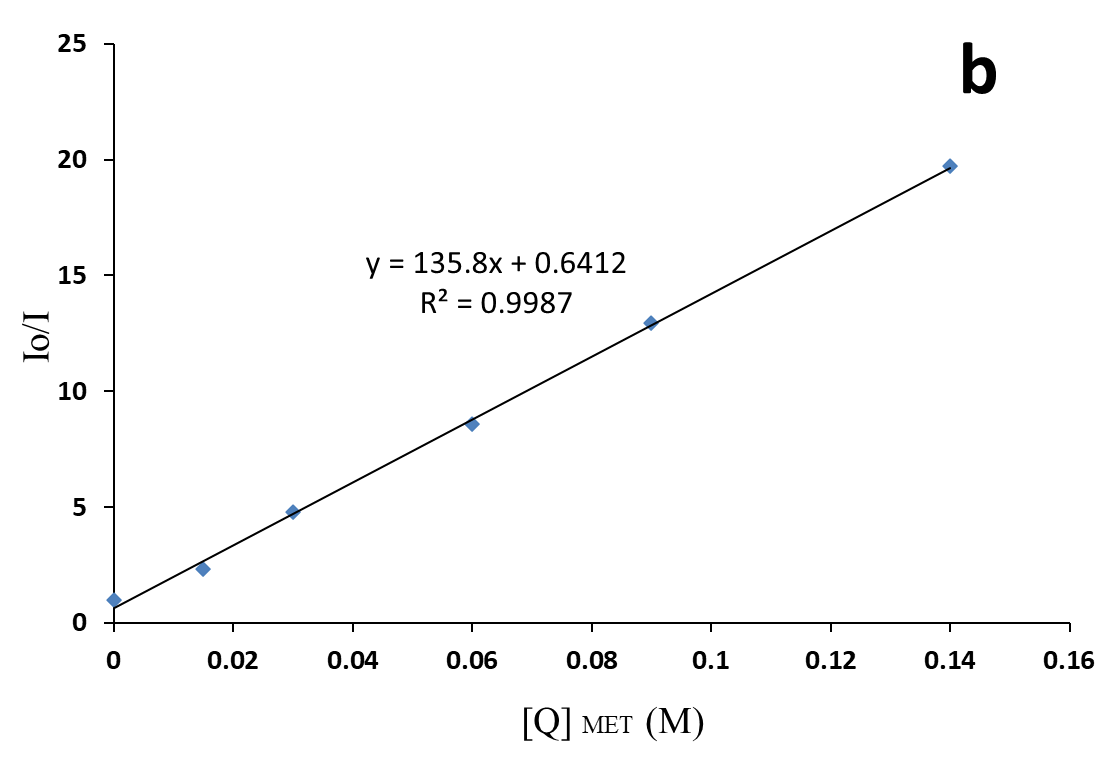


**Figure S8.** Stern-Volmer plot between Io/I and (a) [Q] TET, and (b) [Q] MET.
